# Indigenous institutions as adaptive measures to environmental dynamics: an ethnographic study of Loba Community of Upper Mustang, Nepal

**DOI:** 10.1186/s41257-023-00084-1

**Published:** 2023-03-27

**Authors:** Man Bahadur Khattri, Rishikesh Pandey

**Affiliations:** 1 Associate Professor of Anthropology at the Central Department of Anthropology, Tribhuvan University, Kirtipur, Nepal; 2 Associate Professor of Geography and Environment at Pokhara University, Pokhara, Nepal

**Keywords:** Adaptive measures, Environmental dynamics, Institutions, The Lobas, Nepal

## Abstract

This paper investigates how different institutions of Loba communities of the Upper Mustang work together and facilitate the community to cope with the environmental dynamics in the region. The indigenous institutions are place-based, and their evolution is concerned with reducing vulnerability and enhancing the resilience capacity of place-based communities to cope with and adapt to local natural and socio-cultural environmental dynamics. The paper is based on anthropological fieldwork. Qualitative data were collected by applying observation and interviews. The paper presents the role of the *galbo*, (Lo King), *ghenba* (Village Chief), Lama (Monk), and *dhongba* (Household) as local institutions that act in close relation and make community-level decisions. The findings reveal that the King is seen as the leader whose governance best suited to the local natural environment, cultural practices, and economy. The Lama plays a major role in reinforcing local rules, while the *Ghenba* is an agent who mediates the Lo King and people in materializing rules and operationalizing institutional mechanisms. The *Dhongbas* are units of production of the local social-ecosystem that are entitled to use local resources within the context of the institution’s agreed rules, norms, and values. These local institutions are cooperating well, successfully regulating, managing, and protecting agricultural, forest, and pasture lands, and maintaining the monuments in Lo-manthang for centuries. However, recent social-environmental dynamics such as climate change, migration, and modernization are reducing the relevancies of traditional norms and practices. Nevertheless, the institutions are working hard to continue their existence by frequently modifying their rules and norms.

## Introduction

The adaptive value of local institutions has been widely discussed in anthropological studies since the time of Julian Steward (1972), Marvin Harris during the 1960s and 1970s, Kottak (1980), Rappaport (1999), and Crate and Nuttall (2009). They focused on the role of local institutions in enhancing adaptive capacity to maintain the livelihood system (food, fuel, shelter, health, education, communication system, sovereignty over resources, and their management) in resource-scare ecological regions. Institutions are understood in different ways, however, in social anthropological studies, they are recognized both as formal and informal organization having procedural and functional components of a community, public, civic, and private spheres (Uphoff & Back 2006). Gurung (1996) described a local institution called *satthari* (village council or a structure of village society), which is composed of different clans and kin groups in a community that legitimately works for local jural and political purposes. Ostrom (1990, 1993) has conceptualized eight design principles of local institutions: defined boundaries, provisional equivalence between benefits and costs, collective choice arrangements, monitoring, graduated sanction, conflict resolution mechanisms, minimal recognition of rights to organize and nested enterprises. The essence of religious value with ritual tie-up (Chhetri 2008) and equity among the beneficiaries of the institution (Uprety 2005) are also observed among the Loba of Lo-manthang in their irrigation management, which can be added to the list of design principles for this study that facilitate the local community for enhancing the adaptive capacity of individuals, households, and community. These institutions are built-in social bonds, known as social cement, with reciprocity for mutual benefits (Yaro et al. 2015). The purpose of such institutions is wide-ranged across the communities, from economic, social, cultural, religious, political, and educational to environmental. Agrawal (2008) emphasized understanding such institutions from their roles in shaping adaptation and improving the capacities of the most vulnerable social groups in any social and environmental upheavals. Others also focused on various components of institutions to facilitate adaptation to climate and environmental dynamics: on natural resource management highlighting socio-economic, political, and religious governance, mainly in the forest, pasture (Chhetri 1999; Gurung 1996; Fürer-Haimendorf 1975) and irrigation (Chhetri 2008; Uprety 2005); Nepali (1965) studied *guthi,* a local institution, of Newari people in Kathmandu, Nepal, which has multiple functions in the community.

**Fig. 1:**
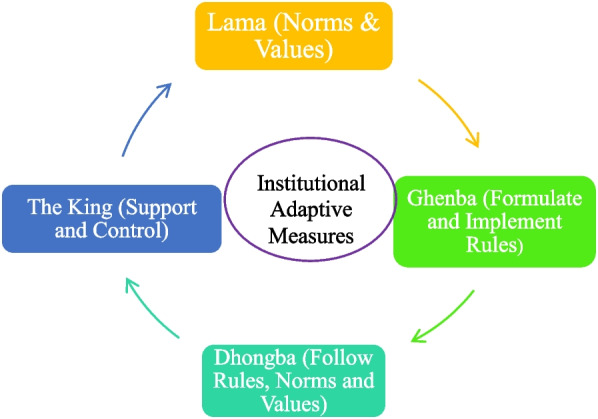
The conceptualization of adaptive measures of Loba indigenous institution

In Upper Mustang, Lo-manthang, different traditional institutions, such as the *King, Lama*, *Ghenba*, and *Dhongba*, prevail and have played different roles in maintaining the socio-political positions of the place, which is crucial in sustaining the environmental as well as the social system. Nevertheless, as the Upper Mustang has experienced rapid changes in socio-economic, political and environmental systems in the last half-century, we investigated their roles in adaptive measures to those changes using the following conceptual framework (Fig. 1).

Figure 1 presents the major local institutions and interconnectedness that collectively sustain the livelihood of the Loba people. The King and *ghemba* formulate or design rules and govern the community affairs, whereas Lama or *gomba* educate people about peace and harmony and ensure justice in society following the norms and values of Tibetan Buddhism. The household (*dhaonba*) is the smallest unit of production of any given local social-ecological system (Pandey & Bardsley 2015), and follows the rules, norms and values prescribed by the other three institutions that sustain their livelihood.

## Methods and data

This paper is a product of multiple fieldworks in different villages of Upper Mustang, Nepal, between 2012 to 2016. The data and information were collected under the PhD research project (Khattri 2021) of the first author. Field observation was one of the principal methods of collecting primary information, followed by Key Informant Interviews (KII). In-depth interviews with 15 farmers, 10 traders, 5 Buddhist monks* (Lamas/* religious leaders), 1 *Amchi* (Himalayan traditional healer), two development practitioners, two local teachers, and two village heads have been conducted by the researcher, along with field research assistants. The semi-structured checklist was prepared to guide the KII to seek information on institutions that govern their unity with the Lo kingdom, craft their norms, values, beliefs, perceptions, and adaptive strategies to cope with the changing socio-economic, cultural and environmental conditions. The selection of the KII was based on informants’ experience and their knowledge of the respective theme of the study. We have also used secondary data to evaluate the facts and ideas, from published scholarly journals to grey literature on the related themes. Primary data were compared and contrasted during the analysis. The fieldwork in Lo-manthang, Upper Mustang started in 2012, but regular interviews and observations were conducted in 2014, 2015, 2016, and 2023 respectively. This study adopted multi-site ethnography, which is suitable in the modern social context, as argued by Marcus (1995). The Loba informants spoke well in the Nepali language, which also made it convenient to do fieldwork much faster.

There are several studies on Upper Mustang, particularly on Lo-manthang. These studies have been carried out from historical, religious and economic perspectives. For example, Chhetri (2008) studied irrigation and highlighted the *ghemba*’s role; Craig (2011) highlighted the Loba migration to the USA; Darnal (2001) focused on tangible heritages while Dhungel (2002) focused on the history of Lo Kingdom. Similarly, Ojha (1986) highlighted economic life; Pissel (1965) presented the general description of Loba from a Tibeto-centric perspective; Sharma & Gurung (2056 BS:^2000^ AD) documented religious sites; and Tulachan (2003) focused on tourism. However, existing literature grossly ignored the value of indigenous institutions as the adaptation mechanism to socio-cultural, techno-economic, politico-institutional, and geo-environmental dynamics.

Anthropologists have focused their studies on cultural ecology and ethnoecology. For them, climate change is connected to several things that include but are not limited to “the nexus of nature, culture, science, politics, and belief” (Barnes & Dove 2015), which helps to highlight contextual knowledge of cultural realities. At the same time, anthropologists are engaged in documenting “indigenous environmental knowledge”, “traditional ecological knowledge”, “indigenous technical knowledge”, “local knowledge”, “folk climate models”, and “cultural-specific knowledge” (Ellen et al. 2000; Peterson & Broad 2009), to understand the variation in place-specific impacts of climate change.

## The identity of Loba community

The issue of identity is complex, multifaceted, and overlapped. This paper presents Loba identity on their self-categorization, language, homeland, cultural practices, religious or spiritual practices, settlement types, types of social categories, and livelihood pattern, because without such knowledge, the roles of their institutions in adaptation facilitation cannot be understood adequately.

There are more than 100 ethnic/indigenous groups in Nepal, which constitute about 35% of the country's total population as of 2011 (Central Bureau of Statistics [CBS] 2012). Lobas are one of them and quite a few in terms of absolute number. Lobas speak their own Loba language, which is similar to the Western Tibetan Language. The Lobas live in Upper Mustang, which is remotely located, highly dynamic, full of environmental diversity, and consists of specific niches to the people (Khattri 2021). The Upper Mustang lies on the northern side of the Mustang district, Gandaki Province of Nepal. Lo-manthang and Lo Ghekar Damodar Kunda are two rural municipalities where thirty-nine village settlements are located (District Development Committee, Mustang, 2068 BS: 2012 AD). The total population of Upper Mustang is 3322, which has the lowest population density (1.49 person /km^2^) in Nepal comparing the national average of 198 person/km^2^ (CBS 2022). As the study site is Lo-manthang of the Upper Mustang, the Population and Housing Census 2011 documented 569 individuals as the area’s total population (CBS 2012).

The Upper Mustang lies between 3000–5000 m from sea level, however, the main human settlement of Lo-manthang is located at an altitude of 3700 m above the sea level. The maximum temperature in June and July reaches up to 23 degrees Celsius and the minimum temperature in January goes down to -20 degrees Celsius. The mean maximum temperature in Nepal increased by 0.06^0^C per year between 1977 and 2000 (Shrestha & Aryal 2011; Shrestha, Gautam, & Bawa 2012). Similarly, the rates of temperature rise vary in different ecological zones of Nepal: Trans-Himalaya (0.09^0^ C), Himalaya (0.06^0^C), Middle Mountain (0.08^0^ C), Siwalik (0.04^0^ C), and Tarai (0.04^0^ C). Upper Mustang is a trans-Himalayan desert (cold) area with an annual average precipitation of less than 20 cm. The area is experiencing a high rate of climate change. The temperature is rising at 0.048 degrees Celsius per year, and the annual rainfall is decreasing by three millimeters (mm) per year (Khattri 2021; Khattri & Pandey 2021).

The trans-Himalayan region of Nepal has a unique natural landscape and is also called a hidden paradise on Earth. The area was forbidden to foreigners until 1992, so it used to be recognized as the ‘forbidden kingdom of the Himalayas.’ The Government of Nepal adopted only the high-yielding and controlled-flow of tourists after 1992. Foreign tourists pay US$ 50 per day to obtain the permit and can stay up to ten days per trip. The Loba settlement is built as a clustered, mud-walled historical town. Some Lobas use caves as houses and monasteries. Since the opening of the place for outsiders, cave settlement has been the point of attraction to tourists. The Annapurna Conservation Area Project (ACAP), a Unit Office at Lo-manthang, shows that a total number of 52,559 foreign tourists visited Upper Mustang from 1992 to 2019.

Archaeological investigation of the region shows that the Loba people have settled in the region and cultivated crops for around 3000 years (Knorzer 2000). The Upper Mustang was established as an independent state by King Amepal in 1440 AD. Until 2008 AD, Lo-manthang was the capital town of the Lo Kingdom and Jigme Dorje Palbar Bista was the last King who had official recognition of *raja* (titular King) from the Government of Nepal. Until 2008, there were four titular kings in Nepal (Mustang, Jajarkot, Salyan, and Bajhang). The Last King of Mustang, the 21^st^ descendant of King Ame Pal—1440 (Dhungel 2002), died in 2016.

The social and cultural hierarchy of the Lo Kingdom is significantly different from the caste and ethnicity system of Nepal. Their hierarchy is not based on untouchability and lacks a higher level of exclusion, even in private spheres. The Loba follow horizontal social stratification by their occupation, educational level, property holding/access, fluency in languages, gender, and lifestyles. Nevertheless, they are categorized as the *Kutak* (Bistas), the *Phalwas* (the Gurung), the *Drokpa* (nomadic pastoralists); and the *Ghara* (occupational groups) based on their blood relation, power position, and occupation. The *Kutaks* include the King and their relatives, who write their surname Bista and are considered the local ruling class and place themselves at the top of the social hierarchy. The *Phalwas* are the most dominant and economically powerful and place themselves in the middle of the social position. The *Drokpa* are nomadic pastoralists and rely on yak, sheep, and goat herding in the highland pastures of Upper Mustang. The *Ghara* is a collective term used for occupational groups at the lowest position in the social hierarchy and the politically and economically weakest sub-group of the region*.* The occupational groups include the *Ghara* (smith), the *Shemba* (butcher), the *Chemba* (leather worker), the *Nepa* (leather processer), the *Samjuwa* or the *Sumbra* (tailor), and the *Emeta* (musician). The *Ghara* settlement lies outside the main settlement in Lo-manthang, which is more or less the same pattern as the traditional Newa: settlements of the Kathmandu Valley (Pandey 2004).

The Lobas categorize themselves into two broad religious and householder’s categories. The first category is *ghiughar chhumbe*/*mhigyaba*, who are engaged in worldly/materialistic life (politically and economically) and the second category is *ranchunchube*/*dhawa* who are engaged in spiritual and religious life. The *ghiughar chhumbe*/*mhigyaba* grow long hair, are marriageable, wear white clothes, and stay in white-colored houses. However, their everyday clothes and hairstyles have changed with the course of modernization. The *ranchunchube/dhawa* is represented by the monastic Lama. They shave their hair, wear red clothes and reside in the monastery, which is red-colored. The color identifies them as the Lamas.

The *ru-shya*, cross-cousin marriage, is also a cultural identity of Lobas. The “*ru*” is understood as bone and related to the father’s line; and *shya* is flesh related to the mother’s line. Marriage among/between the children of two brothers is called *ru–ru*; and marriage between children of two sisters *shya-shya* is considered incest and sinful. For Loba *ru-shya,* marriage relation is perfectly desired, similar to many Himalayan indigenous ethnic communities (Godlstein 1971; Levine 1981; Vinding 1979), including Magar, Gurung, Thakali. However, this practice is being changed slowly as new generations of Loba began to marry outside the cross-cousin relations. The *Loshar*, *Tiji*, and *Yartung* are major seasonal and ritual festivals of Loba that supports maintaining social and cultural relationship among them as well as attracting tourists to Upper Mustang.

Cultural kingship is still functional in every aspect of cultural life in the Loba community. The Lobas speak the Tibeto Burman language and follow different sects of Tibetan Buddhism. The dominant sect is Ngorpa, a sub-sect of Syakyapa. They had practiced polyandry marriage until the recent past. The Loba culture has historical, cultural, linguistic, and ethnic relations with Western Tibetans (Dhungel 2002; Fürer-Haimendorf 1975).

The Loba livelihood includes agriculture, livestock management, trade/seasonal, tourism, and labor migration. They produce single seasonal crops of few varieties (wheat, naked barley, mustard, potatoes, buckwheat, vegetables). The single growing season a year in the region where the minimal size of farmland is available causes inadequate local food production that has challenged food and livelihood security in the region (Pandey 2017).

## Results and discussion

### Environmental dynamics

The people of Lo-manthang have experienced rapid changes in the natural environment, geo-political relations, political changes, infrastructural and technological development, trade, and economy since the 1950s. The major changes observed during this period and their implications in the life of Loba Community of Upper Mustang are briefly discussed below:

### Geo-environmental dynamics

The analysis of meteorological data shows the rise of temperature and decrease in rainfall in the Upper Mustang (Khattri 2021; Khattri & Pandey 2021). The changes are limited to the rise of temperature and the forms and seasons of precipitation (Pandey 2016). The people of Upper Mustang have also perceived the changes, particularly the warming, changes in the forms and seasons of precipitation and changes in wind characteristics such as duration, intensity, and wind -temperature (Pandey 2019). Similarly, the seasonal weather pattern has become unpredictable as some winters are warmer than the springs as winter snowfall is shifted to early spring. These changes have made Upper Mustang even drier since the snow in the spring melts faster, increases runoff, and reduces water storage in the form of snow, therefore decreasing the availability of water but increasing landslides and flooding. Such changes have severely impacted agriculture, livestock, and the mobility pattern of Loba people for livelihoods. Other visible impacts reported are increased invasive species, changed phenology of plants, increased incidents of crops and livestock diseases, and changed habitats of disease vectors. Nevertheless, Loba people are struggling to cope with and adapt to such geo-environmental changes through the adoption of various strategies such as: alteration of crop calendar, improved drainage management and erosion control, increased irrigation and regulated use of water, change in livestock types, herd sizes, and feeding practices, as well as the practice of agro-forestry: grass seedling, fodder, and timber trees plantation (Pandey 2019).

### Socio-economic and cultural dynamics

The Loba people were traditionally practicing a polyandry marriage system which has changed to monogamy. The most important livelihood of Lobas relied on salt (rock salt) and grain trade, which connected them to the central hills of Nepal and India. However, this trade ended totally after the 1950s. The seasonal labor migration from the region started about 50 years ago. Many Loba people migrated to work in high-earning countries and regions like the USA, Europe, and Japan, and supplied a high cash flow. The ever-increasing cash income has a greater impact on the economy of households and communities of Loba. Non-government Organizations (NGO) have contributed to changing people’s coping mechanisms toward livelihood stress. The introduction of tourism after the 1950s partially in the region and openning to outsiders after the 1990s also contributed to understanding the value of their cultural and natural heritages (Tulachan 2003). At present, Upper Mustang is connected by road network to Jomsom, the headquarter of the Mustang district of Nepal. Electricity, telephone and internet facilities are extended to this remote location. Lobas have started using the threshing machine for crop harvest and tractors and trucks to deliver fuel, food, and bring local products to the market. Traditionally, the means of transportation used to be the draught power, particularly the mules and horses to carry goods and oxen for farm-plough. The Government of Nepal contributed a lot to developing a formal education system in the region, where NGOs have also implemented their educational program in coherence with government efforts. NGOs also assisted Gombas financially and materially. These forces contributed to bringing notable changes in livelihoods and food security, household management, community monastery management, dress pattern and food behavior of Loba community (Pandey 2017).

### Political-institutional dynamics

The politico-institutional changes in the Upper Mustang have been remarkable in the last seven decades. The country changed its political status from a unitary Kingdom to a federal democratic republic recently, although major political changes were observed in 1991 and 2006. The Upper Mustang is one of the few areas affected highly by such political change. The Lo King system with the privilege of titular Kingship ended in 2008. Although the informal influence of kingship still prevails in the Loba cultural practices, the political changes in Tibet and the Khampa rebellion, who settled in this region, had larger consequences for Loba’s livelihoods. The area became restricted to foreigners for centuries and opened for controlled tourism only after 1992.

## Institutional adaptive measures

The local socio-economic, political, and environmental conditions have changed in the Upper Mustang in the last seven decades while the Lobas have been coping and adapting to all of these changes, whether well or hard. The Loba local institutions, however, have supported the coping and adaptation of Lobas to the local environment. The Lo King, *ghenba*, Lama, and *dhongba* had a close relationship and interdependency in Lo-manthang until now.

### The monarch as the protector of Loba livelihoods

The Loba King is believed to be the incarnation of Manjushree, the God of knowledge, in the form of the Goddess Saraswati. The King, as an institution, has played a crucial role in establishing, protecting, and promoting Tibetan Buddhism, especially the Sakyapa sect in the Lo Kingdom. The Lo Kingdom and Loba King played a role in achieving and maintaining the autonomy of the region and local resources – land, water, forest, and pasture. The monarch also protects the livelihood of the Loba community by maintaining peace and harmony among the Loba people through formal rules and regulations.

Historical studies documented that the Lo Kingdom was incorporated into modern Nepal in 1789, maintaining a distinct cultural identity. From the time, Mustang became the vassal state of Nepal and the King of the Mustang had the titular privilege of enjoying the title of *raja* (the King) by receiving state grants and customary rights from the Kingdom of Nepal (Dhungel 2002).

The Mustangi King had a good relationship with the Kings of Nepal in Kathmandu. Mustangi Kings were also loyal to the Nepali people. They have historical contributions to promote the Nepali economy and sovereignty of the country. The Mustangi King Gyam Parabal led the Nepal Government delegates in 1895, during the dialogue on the border dispute, with China’s Tibet, related to the salt and grain trade in Mustang. Mustang was close to the Tibetan salt mine of Naithapaila and Lo-Kingdom had a customary monopoly on the salt trade. The Mustangi King initiated the dialogue as some traders were violating the customary monopoly of the salt trade (Uprety 1980). Again, Lo King Jigme Dorje Palbar Bista took the side of Nepal Government during the Khampas rebellion when they were waging war against China from Mustang. Because of the support of the Lo King, it became possible for the Nepal Government that the Khampa rebellion was disarmed in 1974. Although the formal monarchy of the region was abolished in 2008 from Nepal, Loba communities still follow the monarchy institution informally and the King has not lost the respect of the Loba people. The *Kuthak and ghenba* support the monarch in political affairs and ruling the Loba community of Upper Mustang. In this way, the Lobas have adapted the politico-institutional changes by informally accepting the King as their supreme governance institution.

### The Lama: a spiritual educational institution (Tibetan Buddhism) of the Loba community

The Lama, as the chief of spiritual/religious works for the individual, household, and community, also plays a role in the sphere of education and ritual activities. Education and rituals are integral parts of livelihood systems, particularly as the primary function of Buddhist education is to develop an attitude of having belief and values for the individual, nature, and a system of balance, harmony, and co-existence. The non-violence value of Buddhism are transferred to the people via rituals, performances, and direct teaching. The reflection of such teaching can be observed in people’s behavior toward animals and plants. It teaches respect for the diversity in society and nature. Although the Loba follow the Sakyapa sect of Tibetan Buddhism; they are also influenced by the Bon religion as they practiced Bon in the past (Dhungel 2002).

Lo Kingdom continued the practice of cultural systems of resource use and population control. Historical writing (Dhungel 2002) and local oral history information that the Lo region was influenced by Tibetan Buddhism which was introduced by Guru Rinpoche (Padmasambhava), who also established the Lo Ghekar monastery in Marang and Upper Mustang, after the domination of the Bon tradition of the region during 746–47 AD (Dhungel 2002). Furthermore, Amepal, the first King of Lo, played a significant role in the spread of the Lamaism in the Sakyapa sect of Tibetan Buddhism. Building monasteries and Gomba are the founding traditions in Lo-manthang; where three main Gombas, which are about five centuries old, are located.

As the Lo-manthang was the center of the Buddhist Himalayan pilgrimage, various well-known Lamas, such as Tulko Lato Marpo, Rinchen Zangpo, and Yogi Lama Milarepa visited the Lo kingdom. Milarepa Lama stayed one year while traveling to Kailash Manasarovar (Dhungel 2002). This region has also been recognized as a meeting place for Buddhist scholars of different sects, both by native and foreign origin, even from distant places such as Magadha (India), *Simhala* (Sri Lanka), *Balyul* (Kathmandu), *Khache* (Kashmir), and *Bod* (Tibet) (Dhungel 2002). The Lo King and his officials sponsored to arrange religious councils between 1472–1475, where over nine hundred monks attended the meetings (Dhungel 2002). Among the four major sects (Nyingmapa, Sakyapa, Kagyupa, and Gyalugpa) of Tibetan Buddhism, no Gyalugpa monastery was founded in the Lo kingdom.

Lama/monks work to improve the spiritual life and general health of the Loba community. They support people with the spiritual power that they have gained through meditation. Lamas perform all sorts of rites and rituals from birth to death. They perform many rituals at the palace and Gomba (monastery) for the well-being of humans, animals, and plants. According to the Amchi Ghyacho, there are three kinds of human life situations in which a Lama needs to perform rituals: *Nakchi* rituals are performed for sick people; *Sinchi* rituals are performed for dead persons; *Karcha* rituals are performed during the marriage and the birth of a child in a family.

Lamas also play a leading role during disasters and support people in coping. Hari Bahadur KC, then Head Teacher at Dibya Joyti High School Chhoser in Upper Mustang, an eyewitness of the Glacier Lake Outburst Flood (GLOF) in 1984, described the situation in which a Lama led a ritual and walked in front of a ritual procession holding a golden Buddha idol, sprinkling the holy water, and chanting sacred *mantaras* for the peace and security of the people, plants, and livestock during the disastrous GLOF. Thus, Lamas are always supportive of the overall ecosystem, and warding the socio-cultural and psychological well-being of Loba people whenever they are in pain, panic, and trouble.

Lama teaches ordinary people about offering water (*tho*) and light (*chhume*) to God. It symbolizes knowledge and virtue among individuals and family members. They believe that eternity exists in the light, which is achieved after offering it to God. The *chhume,* light supports achieving *shil* (good manner, meditation or concentration, and enlightenment). The process of offering water, flowers, incense, light, fruits, words, and greeting God, ordinary people, animals, and plants have defined ways and manners that should be followed to ensure peace, harmony, and prosperity in the community.

One of the works of the Lama is to assist people in erecting *lungta* in different locations of the settlements, agricultural fields, and sacred sites, including the cemetery. Lama Phunchok said:

The fluttering prayer flags (*lungta*) are a piece of cotton cloth squared in size, with five colors: white, blue, yellow, green, and red. These five colors symbolize wind, sky, earth, water, and fire, respectively. The size of the piece of *lungta* needs to be proportionately equal. This means all the members of households and communities need to have peace and prosperity in a balanced way and they require all elements of nature outside and inside of their bodies.

The Nunnery system is about a hundred years old in Upper Mustang. The girl child is usually entitled to become *Aani*, and they assist in maintaining the Gombas and the Temples. *Aanis* are active in Muktinath temple and are the main actors in controlling or maintenance of the process of worshipping. According to the key informants, *Aani* Gomba was established about a century ago, with the suggestion of a lama from Bhutan. *Aani* Gomba was found in Thinger, Tsarang, and Lo-manthang in the past. However, except in Lo-manthang, there are only a few *Aanis* in Thinger, and Tsarang. *Aanis* were not allowed to marry and had to shave their heads and wear red clothes. This practice was also a part of population control. In the present day, Lobas have introduced monogamy and the family planning system to control the population, which has challenged the traditional lifestyle on the one hand, and on the other, the population of Lamas and *Aanis* is decreasing sharply at Gombas.

### *Ghenba*, an indigenous governing system

The *ghenba*, the village head, is an age-old local institution built upon the socio-cultural and political reality of Loba people and their environmental condition. The *ghenba* plays an active role in agricultural activities, irrigation management system, and the protection of crops and pastures. They develop rules with a collective decision for transhumance practice. They allocate some pasturelands as protected areas and used only in the scare period (February–March) when their livestocks become the physically weakest and most vulnerable due to the shortage of fodder. A similar traditional customary institution holding the administration function and engaging in socio-economic, political, and religious activities is also found in Manang district of Nepal (Sherpa 2015). The village head political system of Lower Mustang is described by Vinding (1994), in which two village heads, a treasurer, and six village workers are elected each year by the villagers, which is different from the formal politico-administrative governance unit of the state. The main function of such indigenous institutions is to formulate plans and supervise public works, programs, and regularly organize communal worship in an appropriate way. They also mediate and hear the local disputes relating to the civil law of the village. The village workers function as watchmen, look after public taps, including whether people use them for washing clothes, as the rules prohibit them from doing so. They are also responsible for the operation and maintenance of grinding mills in the village.

The *ghenba* system has three key positions in Lo-manthang. They are *ghenba*, the village head, *midhi*, the judge, and *chhime*, the messengers/watchmen. Their number varies, such as there are one *ghenba*, two *midhis*, and six *chhimes*, which is more or less similar to the formal governance council at the local unit. They take specific roles and responsibilities of irrigation management and other issues of community spheres. The ghemba has overall led and represented the team, chaired and led meeting(s), his role is to plan farm-related problem-solving and execute the decision made by the council. Among the two *midhis*, one is appointed by the King, and another one is appointed by the village head. The *midhi* is a post that acts like a judge who advises the *ghemba* and the King when a dispute occurs. The six *chhimes* watch the livestock to prevent them from encrouching to farmlands. Their leadership is selected from each household of the *Phalwa* (Gurung) community on the basis of turn system. Chhetri (2008) has called it the reflection of the power structure of Lo-manthang. Amchi Gyatso Bista informed that the number of village council members increases up to eighteen when they need to decide a date for the crop harvesting and electing the next *ghenba*. The roles of *ghenba* system are crucial for supporting agricultural activities, including canal maintenance, water distribution and crop protection from livestock encroachment, and providing justice to the victim.

The *ghenba* system in Lo-manthang is led by eight households from Bista/Kutak clan. A new *ghenba* takes responsibility for one agriculture cycle, which begins from the performance of *sakaluka* ritual and works until the next *sakaluka*. The Loba produces only a single crop in a year, and the tenure of the village head, hence, also becomes one agriculture cycle. The *sakaluka* ritual is the function of worshiping local spirits (*nagas, serpents*) where a lady plows the land at the beginning to mark crop cultivation time (Khattri 2021; Khattri & Pandey 2021).

Anyone who breaks the law and commits multiple offenses such as water theft (diverting the irrigation water to their field without the turn for it) and letting or even driving livestock to the crop fields of the neighbor, and ignorance of the village functionaries, are punishable breach of law. Depending upon the case, the norms defaulter may be asked to be present in front of the village assembly, where, the senior Lama from Chodea Gomba and the Lo King might also be attended. The *Ghenba*, Tamanage Bista said:

the fine locally known as c*hheba*, can be asked in a bigger amount. The payment of the *chheba* becomes higher if someone lets the animal to the crop-field during the night, which can reach as high as NPR 50,000 to 100,000, which is quite a high amount of money.

The fixation of the high amount of fine indicates the value of crops and the sensitiveness of crops for food security. Time factor becomes important because of watching convenience to the assigned individuals.

Irrigation management led by *ghenba* as the local institution has at least ten designed principles in Lo-manthang as presented in Table 1.

**Table 1:** The design principles in irrigation management in Lo-manthang, Upper Mustang, Nepal

SN	Designed Principles	*Ghenba* in Lo-manthang
1	Defined boundaries	Very specific in the command area, position and turns are fixed for the responsibility of the head, and assistant
2	Provisional equivalence between benefits and costs	Proportional distribution of water and cost (labor, material) for maintenance of irrigation system
3	Collective choice arrangements	Collectively decided for amendment of rules, if a disagreement occurs, the final decision is made by the King. The senior Lama of Gomba may be invited during the decision-making process
4	Monitoring	Day and night, by *chhime* in two days turn, during the water pressure /scarcity and time requiring protection of crops from livestock
5	Graduated sanction	Fines are fixed in rules, also forgiveness provision is there and is implemented in the specific context
6	Conflict resolution mechanisms	Two *midis*, a judge, respected/trusted by locals, and familiar with the customary law and who can ensure a rational justice to the village, work for conflict resolution. One *midi* is appointed by the King and another one from the village head. Meetings of the *ghenpa* are held weekly to discuss the issues that emerged and identified. Village assembly and the King are invited, depending upon the types and nature of the cases
7	Minimal recognition of rights to organize	This irrigation system is enduringly autonomous and tied to the local culture, and their livelihoods, no external governing body like a formal local council such as Rural Municipality, the district court, or other rules of the state can influence, except material support
8	Nested enterprises	Single tier but when required, multiple nested enterprises are activated
9	Equity	Allocation of water, information sharing, conflict management, transparency, and accountability in front of the village assembly and the King, which is done before taking the next turn to another group
10	Role of religious value and rituals	*Sakaluka* and *Yartung* are well associated, a wider extent of irrigation as headship takes the responsibility of crop harvest, which checks crop situation and collectively decide the date of harvesting

Source: The village head, a key informant,

Note: Table 1 presents designed principles (1–8) adopted from Ostrom (1993), (9) from, Uprety (2005) and (10) from Chhetri (2008), which are contextualized in the case of Upper Mustang specifically in Lobas of Lo-manthang, Nepal

Recently, new issues have emerged in irrigation management from the people who have migrated to Kathmandu and Pokhara and could not cultivate their lands. They are supposed to contribute compulsory labor to maintain the irrigation system. They are absentee landlords who cannot cultivate their lands but want to retain their land under their ownership. They need to pay a little higher amount to the community for that. This issue has been a subject of public discussion, but not yet decided, said Indra Dhara Bista, the local elite and then member of parliament of the Gandaki Province, Nepal. These sorts of issues are pertinent due to their changes in livelihood strategies. Another issue is that some people have given up crop cultivation, but started fodder and tree farming, demanding a lot of irrigation during high water demand.

In Lo-manthang, a drought-prone region, cultivation without irrigation is impossible. Indigenous irrigation management system is as old as the agriculture system that began in Lo-manthang. The technology used to build irrigation canals is gravity flow supported by walls of stones, wood, and soil. The remains of irrigation canals indicate that irrigation canals broke many times because of floods, and it is very difficult to repair them due to the highly dynamic nature of the slope. However, the *ghenba* system has been able to play a crucial role in mobilizing water users, and is useful in the entire Upper Mustang region to mobilize local people for community development and local, ecosystem-based adaptation.

The Loba follow a collective decision of crop cultivation and harvest. The *ghenba* makes rules and regulations on agriculture and fixes a specific day to begin the cultivation and harvesting of crops. The common people are obliged to follow the rules fixed by the *ghenba*. New voices are raised against these age-old rules. Tamtin Gurung, a local intellectual, said that the system is not favorable to the common people and it has not been harmonized with changing weather system. For him, this system always favors the local feudal and gives them priority to harvest their crops first. He further said:

We are facing challenges of climate change, the temperature is rising, and we are not sure about the weather patterns at present, but the *ghenba* system is controlled by the local feudal without considering the new weather dynamics and life and livelihood of the poor. Local feudal are above the local *ghenba* rules. They harvest their crops first and common people harvest crops later. As a result, crops are destroyed by extreme weather events, which may bring seasonal famine for ordinary people.

New situations such as changes in political, economic, and environmental contexts, have led the Loba people to some troubles in continuing the *ghenba* system.

### Dhongba: an institution of ancestral property holders and community service providers

The *dhongba* is an important institution of Loba that maintains succession and inheritance and manages all ancestral properties. Ancestral properties are only transferrable from generation to generation, mainly to the eldest son, as the family's heritage. This sort of household support management overcomes severe famine and extreme poverty caused by disasters. This type of household also has a compatible practice of fraternal polyandry. The main cause of monogamy is quarrel among the brothers (Gurung 2012) and new family law of Nepal. *Dhongbas* are also obliged to send their second son and daughter to monasteries to be trained as monks and nuns. However, people prefer to give birth to a single child these days, so they would not be obliged to send their loved ones to the monastery. Nevertheless, the process has created a cultural problem in maintaining Gomba since the number of Lama is reduced, which is implicated in the availability of lama for rituals despite their rituals demand many Lamas at a time.

*Dhongba* is similar to western Tibetan regions, which maintain ancestral property system, socio-cultural systems, and prepares household members to participate in political and religious affairs (Goldstein 1971). In Lo-manthang, in particular, and in Upper Mustang in general, households are classified into three types; 1) *Dhongba,* 2) *Ghyanchen* and 3) *Samasume*.

*Dhongba* households are ancestral property holders, which is transferred from generation to generation, mainly to the eldest son. *Ghenchen* household is a split from the *dhongba*, often recognized as parents’ household*. Samasume* is another type of household that is established independently. *Samasume* includes those households formed by children who are usually grown up in maternal homes and are illegitimate for the ancestral property. Other household types, such as *phorang* (of a singled man) and *morang* (of a singled woman), are also observable in the place.

*Dhongba* households do have certain community obligations such as maintaining *Gomba* (monastery) (support rituals with food, labor, materials, and fuels), organizing community festivals like *Yartung*, *Tenchi Chiram* (*Tiji*) rituals, and supporting the royal palace as required. They are also required to manage the household economy (land, livestock, trade), and look after their entire family members, including the parents, brothers, and sisters, until they become adults. *Dhongbas* work for the King during crop cultivation and harvesting time. In the past, they were obliged to perform all sorts of work, cooking, cleaning, collecting dung or fuel wood, and fetching water for the monastery and the palace. These tasks used to be performed in the form of *reme ghapkin*, the turn system, particularly while working for the Gomba and the palace (Table 2).

**Table 2:** Rotational division of labor of *dhongba* to serve the royal family in Lo Kingdom

SN	Household Types	Role of Households
1	*Shikira*	To manage firewood/fuel
2	*Chhubindra*	To look after horses
3	*Aamchi*	Amchi Gyatso Bista, the local traditional medicinal healer also the royal priest to perform rituals to royal ancestors
4	*Chhaju*	To look after property/store
5	*Semba Dhonba*	To handle animal butchering
6	*Sibun*	To deliver information
7	*Chhimba*	To irrigate field
8	*Lochhin*	Religious storyteller
9	*Nejuma*	Preparing Tshampa

Source: Key Informant Interview, 2015

*Dhongba* also manages the royal herds. In lieu of their services, King has provided highly fertile land to *dhongbas*, as Funjok Gurung (39) told us that *dhongbas* are entitled to become village chiefs. They are privileged to participate in rituals and community festivals and have influential roles in community decision-making.

Goldstein (1971) has documented that *dhongbas* are economically wealthy and are regarded as more prestigious than others in Tibetan society. They hold sizeable agricultural land from their lord. They are the political authority as well. To receive the land, they fulfilled extensive obligations such as free labor of men and animals and contribution to the palace, both in cash and kind.

## Reme gyapkin, the turn system

**Fig. 2:**
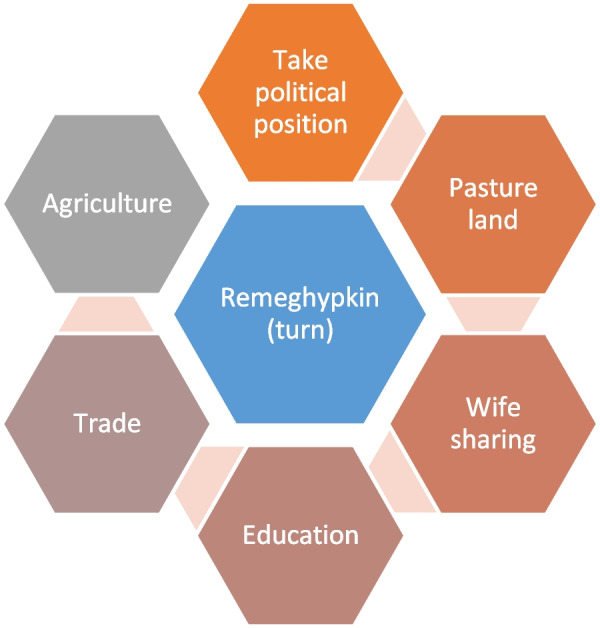
The Reme gyapkin, the turn system followed in different spheres of Loba life

The *Reme ghyapkin* is a system of sharing and managing resources, costs of community work, and benefits of community utilities on a rotational basis. It is multi-sectoral practice (Fig. 2). It operates at different levels, from individuals to households, clusters, and in broader areas such as region. The main function of this system is to prevent conflict and violence among the main competitors/stakeholders in resource and service use. The Loba practice this system wherever they go and whenever they need to share something. This system was followed also in Varanasi India during winter trade.

The turn system is widely followed in different spheres of Loba life. In agricultural land, black crops and white crops are cultivated annually and are the alternative crops. The annual turn is followed in traditional leadership (*ghenba*, *midhi*, and *chhime*). Pasture is managed in turn by splitting them into plots. Polyandrous brothers share a wife by turn, as managed by the wife. Education and trade also take turns seasonally.

This turn system is unique to the Lobas and can be considered a primary democratic mechanism, which is instrumental to governing the people of Upper Mustang, particularly in Lo-manthang. This system is practiced in different aspects of life, such as selecting leadership (except the King), distribution of irrigation system, during the *Yartung* taking a turn to follow King’s possession that leads the masses on horse parade, using the public places such as monasteries and Gomba during the ritual performance and feast. In the past, the Loba followed the *reme ghapkin* to serve the palace’s kitchen management, rituals, water, fuel, fodder, livestock, security, and handling other business as well (Table 2). In the polyandrous family, male members follow the *reme gyapkin* system to maintain a harmonious relationship among the brothers in the household. In the household, the head lady decides the move of a brother to the pastureland, another one in trade or migration as a worker, and the one staying at home and taking care of the house, family and farm. Most of the time, the eldest brother remains at home.

Two households were responsible as *nechhang nerpa,* the economic manager of the palace during the King’s rule. Each year, they come from the *Kuthak* family. They arrange all the business and support to strengthen the economy of the palace. To support the palace, some households were categorized as the *nagjin* to work inside the palace: cleaning rooms, carrying loads, processing and cooking foods, and grinding or preparing *tsampa* were their main tasks. Altogether, there were 18 households, they worked on a rotational basis. Each household had a turn of 7 days. This sort of process ensured the access of ordinary household family members to the palace on the one hand, and reduced the hegemony of few people that is observable in formally-employed staff in a formal governance system on the other.

Fifty-three households were the *makhiu*, i.e., the King’s soldiers. They work in the royal fields and look after the royal herd on a turn basis. Five households were *sinkhor*, who were responsible for supplying fuel wood or fuel dung to the palace. According to a key informant, compared to other households, *makhiu* and *sinkhor* received good-quality land from the King for agricultural purposes.

## Role of local institutions in changing socio-economic, and environmental context

**Fig. 3:**
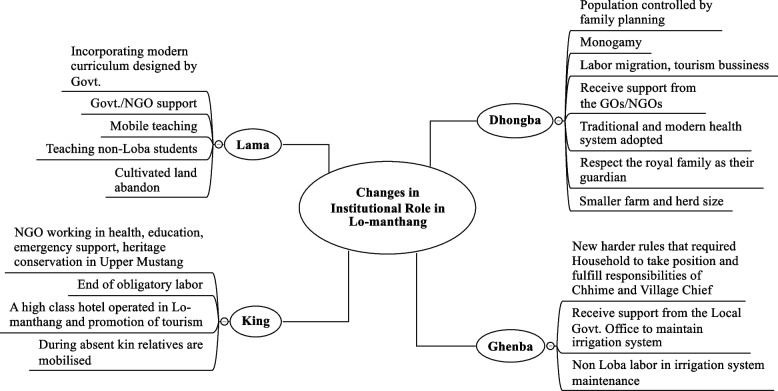
Changes in institutional role in the changing socio-economic, and environmental context

The continuity and changes, and the turn systems are the major mechanisms adopted by the Loba to cope with changing socio-economic, and environmental conditions. Lobas have maintained some traditional practices of livelihood and added new livelihood strategies. The above-mentioned institutions have significantly played a role in the changing context, which is summarized in Fig. 3 and described in the following section.

Figure 3 presents how the traditional local institutions have adapted to dynamic context and maintain the relationship between the people and the local environment. Basically, mobility, external assistance, acceptance of provisions of the Nepal government, democratization, and reform in cultural practices are major strategies in changing institutional dynamics.

### King’s role and the changing socio-economic, and environmental context

The formal Kingship ended by the Nepali state in 2008. The Loba people informally still recognized the former prince as the King. Just a few years back, the King used to organize a feast at the time of Loshar (the New Year Festival) for all the Loba people in Lo-manthang. On such occasions, the Loba people too, used to greet the King with some money and *khada* (a piece of special cloth as the symbol of well-wishes) to the Loba King and the Queen. However, this tradition has seemingly ended recently, as the King and Queen live in Kathmandu because of their fragile health condition.

Obligation to labor support to the King also ended, since most of the cultivated land belonging to the King has been abandoned, and only about a tenth of the total farmland under the former King’s entitlement is cultivated. Amchi Gyatso Bista, a local traditional medicinal healer, royal ancestral priest and former *ghenba* said that the present King was asked if local people should support first to collect fodder from his field and harvest crops by residents, but the King denied to receive support from local people as he would hire labors. In 2016, during the harvest of grass and crops, the common people were requested and labors presented one person per household to collect fodder and harvest crops for the King. At that time, the workers received good food while at work and also received normal wages of NRs. 300 per day per person. In the past, such labor contributions by people to the King’s farm used to be obligatory and free. Local people remembered the past and said that a *knombo*, the basket was torn-off, as they worked at the royal field and palace each year. The change from obligatory labor to paid labor has been witnessed as the titular kingship ended, the last King died; and the family of the present King lives mostly in Kathmandu.

Although informal, the King still has some power, recognized by Loba communities, to modify individual’s or household’s fine fixed by the *ghenba* system. The King’s welfare support to the people, which is implemented through the Jigme Foundation, is mostly directed towards the health sector, daycare centers for children, and the promotion of education. The Jigme Foundation also supported local people through food items, such as rice, salt, and other materials, during the COVID-19 pandemic and associated lockdown, with the support of the local representatives of the foundations, who are from the King’s close relatives.

King’s role has been positive in opening tourism in Upper Mustang since 1992 and the family has also been involved in the tourism sector. They began with running a travel agency and network building with tourists, and also running a curio shop in Lo-manthang, and recently they have been running a hotel for high class tourists in Lo-manthang. This hotel supports to employ some Loba youth and contributes to the economic development of the region.

### Gomba in Lo-manthang facing danger of collapse

The traditional role of the Gomba in the Loba community, as described above, is an institution that maintains a peaceful and just society, as per the values of Tibetan Buddhism. *Gomba* had been playing a role and supportive in controlling the Loba population through promoting celibacy by Lama and *Anis,* which was obligatory to each family that a second child be the *Lama or Anis*. *Gombas* were supported by local *dhongbas*, who were mobilizing local resources. The *Lama* and *Aani* were taking mostly spiritual knowledge and practices from Gomba. This institution was the center of attraction in the past. *Gombas* are still playing their customary role even in a recently changing context where the modern education system has already entered the Loba community, and there is no obligation these days that the second child of the family becomes the Lama or Anis. Furthermore, due to the out-migration of the Lamas and other people, increased dependency on the market and changing livelihood options of the younger generation are also observable in the Loba community. Furthermore, external financial dependency is recognized as a sign of threats to the collapse of *Gomba* in Lo-manthang. The result is that *Gomba* was compelled to modify their existing rules to make people more obliged to support Gomba, particularly the operation of agricultural activities in the land plots owned by Gomba. Lama Phuntchok said:

Chhode Gomba owns big plots of agricultural land, which were to be cultivated by those people who do not produce enough food for their families from their land. The Gomba used to get some grains from the tenants/cultivators as rent, in proportion to the amount of seed being sown in the field. This practice used to be called *phutok*. In the past, Loba had a practice of *bhogmo* which means a half-share of the production to be given to Gomba as the rent for cultivating the land.

Since Loba was compelled to produce grain to sustain their family, the agricultural land had a competitive use. However, as Loba people prioritize cash income away from agriculture, the practice of half-share crop production system hardly exists. People are not even interested in *photok* because agriculture production demands more inputs than outputs, and lacking household labor increases the cost of production further. Additionally, managing the irrigation system has become a big challenge because the Upper Mustang region is becoming drier every year (Khatri & Pandey 2021). Earlier, the land of the Gomba used to be cultivated by those households who sent their children to the Gomba as Lama or Anis. However, as people are no longer interested in educating their children as monks, most of the cultivable lands are abandoned. The Lamas who live at Gomba get support, food, and other things, from the American Himalayan Foundation. The consequence is increased external dependency rather than adapting to the local environmental system.

At present, the ways of life of Lama have changed greatly and those living, studying, and teaching at Chhoede Gomba have a well-off life. We can also observe a new development in the family and marriage system, people are practicing family planning, which allows the birth of a few children, so no more celibacy is required to control the population. This new phenomenon harms Lamaism and monastic life in Lo-manthang. Lama Chhiring Tashi said that promoting family planning and wider advocacy of/for human rights are responsible for decreasing the number of Lama in Chhode *Gomba* in Lo-manthang. Due to the practice of family planning, there is little chance of having a third male child, as a result, the families do not want to separate their second child as well. The local customary laws are obliged to send a second child to the *Gomba*, but when there is only one child, it is not compulsory and customary law is also eroding with the introduction of formal laws such as fundamental human rights. Those people who have traveled outside of Lo-manthang for work, study, or business and have already earned a good amount of money can contribute to rituals. Some Lamas, who moved out from the *Gomba*, run private boarding schools with the support of local and foreign well-wishers. In Lo-manthang, an Amchi School is also established aiming to provide modern education and promote a traditional healing health system. The individual students are also sponsored, mostly by foreign donors. Many young Lamas are giving up their monastic life and starting married family life and the number is growing every year in Lo-manthang. This is happening despite the availability of all sorts of facilities at Gomba. Many people are not attracted to getting involved in ritual activities. Young Lobas are attracted to the modern knowledge system and go to high-earning countries, which is a big challenge to maintain the indigenous knowledge system and the cultural identity of the Loba.

The pressure on monks at *Gomba* from local people has decreased. Now Gomba has started enrollment of novices from other regions of Nepal. Nowadays, weak and poor people from anywhere in Nepal can send their children to *Gomba* in Lo-manthang. These children are mostly sponsored by donors. In the past, rich and well-off *dhongba* families used to send their second son and daughter to the *Gomba*, who were contributors to *Gomba.* However, nowadays, people see other socio-economic opportunities instead of just being a Lama. Nevertheless, some households are bound by the customary rules developed in the past and are still sending their second son/daughter to the *Gomba*.

### Loba migration and challenges in the *ghenba* system

Many farmers have stopped farming activities in Lo-manthang, as a consequence, widespread abandonment of the cultivated land is observed. Amchi Ghyatso Bista estimates that only one-tenth of cultivated land is under cultivation now. Lack of household labor or their engagement in better-paid jobs outside of the village (mostly abroad) and increasing wages of farm laborers further raised the cost of agricultural products. The farm laborers come from the western part of Baglung, Rukum and Rolpa districts of Nepal. As local youth of Lo-manthang migrated out, the cheap labor from other districts immigrated to the place seasonally, mostly to work in farm-fields, repairing irrigation canals, and fencing farm-fields, as well as the construction of house building and roads and bridges. The construction project contractors of the village municipality for developmental works supply the laborers from those districts where poverty and unemployment are high. Village municipality developmental works are mostly done by these in-migrant workers since the members of the Loba community are not much interested in such labor works. Amchi Ghyatso Bista further adds that after April and May, more in-migrant workers than the people of the Loba community can be seen in Lo-manthang.

Lo-manthang Rural Municipality (LRM), the local government provides grants to the *ghenba* system to maintain irrigation canals. As a result, members of the system are less burdened financially. However, a new problem in the *ghenba* system has emerged since some Loba households are not interested in taking responsibility of *ghenba* and *chhime*. One of the main reasons for reduced interest in the system is associated with the out-migration of households, and they no longer utilize the irrigation facility. To overcome this problem, the community imposed a new rule that each Bista household must perform the role of *ghenba* on their turn. Otherwise, that household must pay NPR 40,000 as a fine. Furthermore, taking responsibility of *chhime* becomes more challenging since they imposed NPR. 20,000 as a fine for those households unable to take responsibility of *chhime.* In such situations, the *ghenba* system looks for another person/household who takes the responsibility and pays that money to the person/household. Nowadays, another challenge emerged that many people are willing to pay money instead of playing the role of *chhime.* To cope with this sort of crisis, the *ghenba* System enforced a new rule that every household must play the role of *chhime* and *ghenba* on their turn.

### Community role of dhonnba in the changing context

The Loba community sustained their livelihoods through the adoption of agriculture and livestock system, as well as the salt, and grain trade. These options have changed dramatically in the last few decades with the influence of Nepali state and globalization as a whole. Nevertheless, the Loba community has benefited from this sort of new development in the Lo region. Their life has become much more comfortable in this harsh environmental condition and the impact of modernization is to be prized. Road networks have linked them to the market, which supplies required materials at a lower price and through easier ways than producing those consumables locally. Community support from the formal Government institution and NGOs decreased the role and responsibilities of traditional institutions toward community work. Despite the infrastructural development in the region, Loba has migrated to other places for better opportunities as their traditional livelihood system could not support coping with people’s desires since they are searching for freedom of choice. The *dhongba* households, however, still have certain community obligations such as maintaining *Gomba* (monastery); support in rituals with food, labor, materials, and fuel; organizing important community festivals/rituals like *Yartung*, *Tenchi Chiram* (*Tiji*); and looking after their entire family members, including the parents and brother and sisters until they become adults. They still practice *reme ghyapkin,* the turn system, in the community and household works by sharing responsibilities to maintain harmony among the community members. In this way, the local institutions are supporting Loba communities to adapt to the changes, although the level of success in adaptation in a traditional way is limited, so the institutions, not only the community, demonstrated their dynamism to adapt to the change.

At present the *dhongba* system is facing several challenges because of the changes in the marriage system and childbirth control, as well as changes in environmental and resource conditions. They have become less dependent on the parental property as they have diversified their livelihood system, including but not limited to labor migration and engagement in trade and commerce, including in the tourism industry. They have also explored new livelihood opportunities, which are the outcomes of education and the changed legal system of the Government of Nepal. *Dhongba* was a real-life saver of the household in the past, which, with the course of modernization, their burden has been notably reduced and responsibility has been changed as many Loba households sustain on their own.

## Conclusion

The Loba institutions have enduring characteristics which have facilitated the enhancement of the adaptive capacity of the Loba people in the harsh environmental condition of Upper Mustang. The autonomous institutions of Loba have been playing a crucial role in framing compulsory obligations, ensuring access to and control over valuable local resources through a system of *reme ghapkin,* a democratic system of turn, which is based on community rules. This is a crucial aspect of niche construction based on equality, focusing on irrigation of agriculture fields and management of pasture land, mostly during scarce periods. Despite the changes in the natural environment, geo-political relations, politics in Nepal, infrastructural development, and technological advancement that have taken place within the last seven decades in the region, the King, *ghenba*, *dhongba*, Lama have been playing a crucial role in maintaining a balanced relationship between people, surrounding environment and livestock, which ultimately supports peace, prosperity, and harmony among the people by regulating, managing, and protecting agriculture, pasture, and the monuments in Lo-manthang. These local institutions are altering traditional agricultural practices to respond to the impacts of changes in the physical environment. Indigenous knowledge-based systems of farm, labor, and resource management, accompanied by changes in food preference (food value) and linking agriculture with spiritual practice, followed by occupational modernization and migration, are a high priority in the adaptation process (Khatri & Pandey 2021) that the Lobas have demonstrated through their indigenous institutions. Nevertheless, despite all socio-economic and political development, migration has become a big challenge to continue the role of traditional institutions of the Loba community in Lo-manthang since the increased scarcity of human resources to perform community responsibilities is observed. Some adaptation strategies such as mobility, external assistance, acceptance of provisions of Nepal government, democratization, and reform in cultural practices are found as the major strategies in changing soci-economic, political and environmental conditions. However, Loba people have been adapting to new and heavier conditions by hiring paid laborers supplied from outside. This study has contributed significantly to the advancement of the Ostrom’s (1990, 1993) eight design principles and made it ten, in the case of local institutions of the Loba community of Upper Mustang.

## Data Availability

The data are available.
